# The usefulness of the follicle-preserving sign in differentiating between benign, borderline, and malignant ovarian tumors on magnetic resonance imaging

**DOI:** 10.1007/s11604-025-01905-3

**Published:** 2025-11-10

**Authors:** Daiya Takekoshi, Ayumi Ohya, Takuya Fukuzawa, Mana Maehara, Akira Yamada, Yasunari Fujinaga

**Affiliations:** 1 Department of Radiology, Shinshu University School of Medicine, 3-1-1 Asahi, Matsumoto, 390-8621 Japan; 2 Department of Medical Data Science, Shinshu University School of Medicine, 3-1-1 Asahi, Matsumoto, 390-8621 Japan

**Keywords:** Follicle preserving sign, Ovarian tumors, MRI, Grading, Malignancy

## Abstract

**Purpose:**

The study aimed to evaluate the incidence and features of the follicle-preserving sign (FPS) and investigate its usefulness in differentiating the grades of malignancy of ovarian tumors using magnetic resonance imaging (MRI).

**Materials and methods:**

This retrospective study examined 234 patients of reproductive age with a confirmed diagnosis of ovarian lesions at Shinshu University Hospital between January 2014 and December 2023. Preoperative MR images of each patient were independently evaluated by two radiologists. First, 20 patients with malignant ovarian tumors were randomly selected. To define the MR findings of normal follicles, we observed the contralateral unaffected ovary. Next, the presence of FPS in the affected ovaries was examined in all patients. Finally, the FPS frequency was statistically analyzed in relation to patient age, maximum tumor diameter, tumor malignancy, MRI magnetic field strength, MRI slice thickness (MRIST), and histological subtype.

**Results:**

Among 276 ovarian lesions (benign, 146; borderline, 51; malignant, 79), the follicle-preserving sign (FPS) was significantly more frequent in benign tumors than in malignant tumors (*p* < 0.001), while no significant difference was observed between borderline tumors and the other two groups. Multivariate analysis identified patient age, tumor diameter, and MRIST as independent factors associated with FPS, with MRIST exerting the greatest influence (OR = 0.75, *p* = 0.006). Compared with malignant tumors, benign tumors tended to be smaller and were imaged with thinner slices, contributing to a higher FPS incidence. Importantly, even after stratification by MRIST, benign tumors consistently showed a significantly higher FPS rate than malignant tumors across all subgroups (*p* < 0.05). FPS frequency also varied by histological subtype, being frequent in serous/seromucinous borderline tumors and immature teratomas, but uncommon in mucinous tumors irrespective of malignancy status.

**Conclusion:**

The incidence of FPS was significantly higher in benign tumors than malignant tumors. However, it should be noted that FPS expression is affected by MRIST.

## Introduction

Magnetic resonance imaging (MRI) has become an indispensable tool for the evaluation and characterization of ovarian tumors due to the excellent soft-tissue contrast and multiplanar imaging capabilities of the modality. However, several factors make the accurate diagnosis of ovarian tumors using MRI highly challenging.

Ovarian tumors represent a broad spectrum of conditions, including benign, borderline, and malignant, each presents distinct imaging characteristics [1–4]. However, the overlapping features of these categories often complicate their differentiation. The signal intensity on T1- and T2-weighted imaging (T2WI) can vary significantly depending on the histological composition of the tumor, including fibrous tissue, hemorrhage, or necrosis. This wide variability often demands careful interpretation to prevent misclassification. Thus, the differential diagnosis of ovarian tumors using MRI proves challenging. However, the introduction of the Ovarian-Adnexal Reporting and Data System (O-RADS) MRI in recent years has made it possible to estimate the grade of ovarian tumors more effectively, despite the inability to determine their exact histology.

The follicle-preserving sign (FPS) refers to the normal follicle structure preserved on the margins of tumors that is recognizable on computerized tomography (CT) and MRI in patients of reproductive age [5]. FPS is observed in benign and small malignant/metastatic ovarian tumors, massive ovarian edema, as well as borderline and small round cell tumors of the ovary, including malignant lymphoma and chloroma [5–8]. Among these applications, the usefulness of the FPS in the diagnosis of malignant lymphoma has been particularly noted. In contrast, other malignant tumors are believed to obscure or disrupt the visibility of normal ovarian follicles [9]. However, to date, no detailed studies have reported the relationship between FPS and malignant ovarian tumors other than malignant lymphoma, or its potential role in grading ovarian tumor malignancy.

Hence, our question remains whether FPS could truly serve as a tool for assessing the malignancy of ovarian tumors as previously suggested. Therefore, this study aimed to analyze the relationship between FPS and the grade of ovarian tumors, as well as to elucidate the factors associated with the presence or absence of FPS.

## Materials and methods

### Patient population

This study was reviewed and approved by our Institutional Ethics Committee (approval no. 6077). The need for informed consent was waived due to the retrospective nature of this study.

**Fig. 1:**
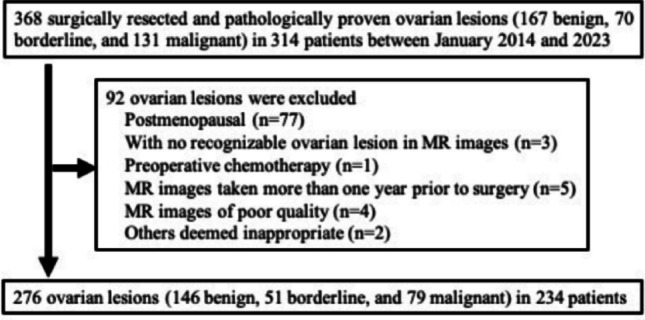
Flow diagram of the study’s inclusion and exclusion criteria. A total of 234 patients (benign, n = 146; borderline, n = 51; and malignant, n = 79) were selected as clinical research cases

Patients diagnosed with ovarian tumors between January 2014 and December 2023 were identified using the electronic medical records at Shinshu University Hospital. We reviewed the pathology database of our institution and selected patients with “ovarian tumors” as the search criteria. The study included patients with ovarian tumors who underwent surgical treatment during their reproductive years (between menarche and menopause). In patients with bilateral ovarian lesions, each lesion was included in the study. All patients had undergone preoperative MRI scans, including T2WI performed, and had a confirmed final pathological diagnosis. We excluded postmenopausal patients, those with ovarian lesions not visible on MRI, patients who had received preoperative chemotherapy, those whose preoperative MRI was performed more than one year before surgery, cases with poor-quality MR images, or those deemed unsuitable for analysis by the observer. The patient selection flowchart is illustrated in Fig. 1.

### Overview of MRI findings and their analysis

All MR images were acquired using 1.5 or 3.0-T scanners (Philips Electronics N.V., Amsterdam, Holland; GE HealthCare, Chicago, Illinois, USA; Canon Medical Systems Corp., Tochigi, Japan; Siemens Healthcare Diagnostics, Erlangen, Germany).

All images were assessed by two radiologists with 7 and 21 years of experience. The radiologists independently evaluated the T2WI of the horizontal, sagittal, and coronal sections, corresponding fat-suppressed images, T1WIs, contrast images, and diffusion-weighted images with apparent diffusion coefficient maps. The reference images were a mixture of 2D and 3D images. The minimum MRI slice thickness (MRIST) of T2WIs for each case is summarized in Table 1.

**Table 1:** Number of cases by the minimum MRIST of T2WIs

The thinnest MRIST (mm)	Number of lesions (%)
1.25	3 (1.1)
2.00	90 (32.6)
2.20	4 (1.4)
2.30	3 (1.1)
2.40	1 (0.4)
2.50	28 (10.1)
2.80	3 (1.1)
3.00	45 (16.3)
3.30	1 (0.4)
3.50	3 (1.1)
4.00	6 (2.2)
4.50	2 (0.7)
5.00	43 (15.6)
6.00	27 (9.8)
6.50	9 (3.3)
7.00	8 (2.9)

MRIST, Magnetic resonance imaging slice thickness; T2WI, T2 weighted image

The definition of follicle cysts in this study was determined through the following procedure. To establish a definition of normal follicle cysts, we first analyzed normal ovaries of 20 patients with unilateral malignant ovarian tumors randomly (patients with bilateral ovarian tumors were excluded). And the MRI findings of the follicles were defined based on the imaging features of the contralateral normal ovary. Histopathological examination confirmed that these 20 patients had normal contralateral ovaries, with no detectable lesions. Two radiologists selected the smallest cyst structure in each of the 20 ovaries. They then evaluated all 20 cystic structures, focusing on the size, shape, wall characteristics, and internal signal intensity of their contents. Then, a structure was defined as a follicle if it exhibited imaging features common to all 20 cystic structures. Next, they assessed the incidence and characteristics of FPS in all lesions based on this definition. They confirmed the reproducibility of follicular cysts in at least two imaging planes on T2WIs. Without referring to the patients’ clinical or pathological information, they evaluated the MR images of the lesions, focusing on the following findings: maximum lesion diameter and the presence of follicle cysts. The maximum diameter of each lesion was measured on T2WI. In case of disagreement, a consensus was reached through discussion. We subsequently analyzed the associations of the FPS positivity rate with ovarian tumor malignancy, patient age, tumor size, MRI magnetic field strength, MRIST, and histological subtype.

### Statistical analysis

Fisher’s exact test was used to compare categorical variables, including the availability of FPS and the categorized numbers of FPS cysts. Moreover, The Mann–Whitney U and Kruskal–Wallis tests were employed to compare continuous variables such as patient age and maximum tumor size across different groups (benign, borderline, and malignant). Binomial logistic regression and multiple regression analyses were utilized for multivariate analysis. Specifically, binomial logistic regression was used to identify factors influencing the frequency of FPS, with the results reported as odds ratios (OR) and 95% confidence intervals (CI). Multiple regression was then performed to determine the factors that influenced the MRIST, with the results reported as partial regression coefficients and 95% CI. In the univariate and multivariate analyses, statistical significance was set at *p* < 0.05. Cohen’s kappa coefficient was calculated to evaluate the inter-reader agreement of FPS. A kappa value of ≦0.20 indicated poor agreement; 0.21–0.40, fair agreement; 0.41–0.60, moderate agreement; 0.61–0.80, good agreement; and 0.81–1.00, excellent agreement. All analyses were performed using SPSS Statistics software (version 28; IBM, Inc., Chicago, IL, USA).

## Results

Based on the inclusion and exclusion criteria, 234 patients (mean age, 40 years; range, 14–54) were enrolled. Bilateral tumors included benign (n = 31), borderline (n = 4), and malignant (n = 7) cases. The total number of ovarian tumors included was 276 (benign, n = 146; borderline, n = 51; and malignant, n = 79). The median age of the patients was as follows: benign, 36 years (range, 14–54); borderline, 38 years (range, 17–54); and malignant, 46 years (range, 18–53). Regarding the analyzed images, the median interval between preoperative MRI and surgical resection was 65.5 days (range, 0–364 days) in the benign group; 63.0 days (range, 18–157 days) in the borderline group, and 45 days (range, 0–207 days) in the malignant group. Among the patients, 172 and 62 patients underwent scans using 1.5 Tesla and 3.0 Tesla MRI scanners, respectively. T2WIs were acquired using 2D and 3D sequences in 273 and 3 cases, respectively.

**Fig. 2:**
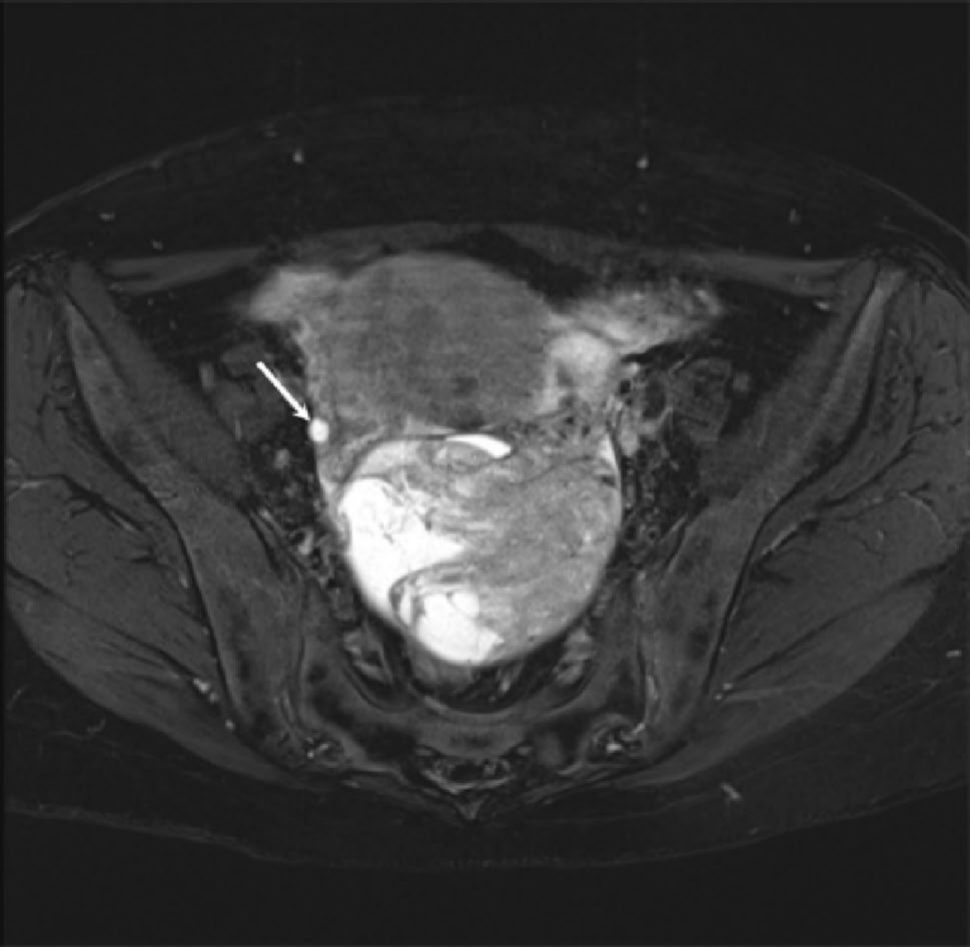
Definition of normal ovarian follicles. A 46-year-old woman with endometrioid carcinoma. A white arrow shows normal follicle on unaffected right side of malignant ovarian tumors in fat-suppressed T2-weighted image. A follicle is oval unilocular cystic structure with smooth wall, and has cystic content with isointense to water

Among the 20 patients analyzed to define the MRI features of ovarian follicles, the median age was 45.5 years (range, 25–52 years). The mean diameter of the smallest follicles in each ovary, across 20 cases, was 6 mm (range, 2–14 mm). All normal follicle cysts met the following criteria on MRI: they appeared as unilocular cystic structures measuring 2–14 mm in size, with smooth walls and a round to oval shape. The cystic contents demonstrated signal intensity similar to that of water (Fig. 2). These characteristics were collectively defined as FPS.

**Fig. 3:**
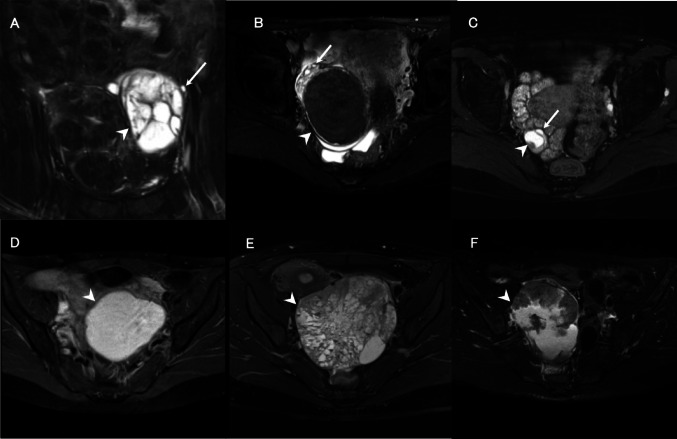
Follicle-preserving sign (FPS) cysts in each pathological group. **A** A 54-year-old woman with mucinous cystadenoma. A coronal fat-suppressed T2-weighted image (T2WI) reveals a follicle cyst (white arrow) at the margin of a multilocular cystic mass (arrowhead) with hyperintensity. **B** A 36-year-old woman with a mucinous borderline tumor. An axial fat-suppressed T2WI displays follicle cysts (arrow) at the margin of a multilocular cystic mass (arrowhead). **C** A 38-year-old woman with endometrioid carcinoma. An axial fat-suppressed T2WI displays follicle cysts (white arrow) at the margin of a unilocular cystic mass with a mural nodule (arrowhead). **D** A 38-year-old woman with an endometriotic cyst. An axial fat-suppressed T2WI demonstrates no follicle cysts at the margin of the unilocular cystic mass (arrowhead). **E** A 44-year-old woman with adult-type granulosa cell tumor. An axial fat-suppressed T2WI reveals no follicle cyst at the margin of the sponge-like mass (arrowhead). **F** A 45-year-old woman with serous carcinoma. An axial fat-suppressed T2WI displays no follicle cyst at the margin of a unilocular cystic mass with a solid component (arrowhead)

FPS was detected in 143 of the 276 lesions (51.8%). The incidence of FPS was significantly higher in benign tumors than in malignant tumors (*p* < 0.001). No significant difference was observed in the incidence of FPS between the borderline and the other groups (Table 2). The inter-reader agreement of the FPS between the two radiologists was good for benign and borderline ovarian tumors, and moderate for malignant ovarian tumors; the Cohen’s kappa coefficients were 0.711 for benign ovarian lesions, 0.720 for borderline ovarian tumors, and 0.574 for malignant ovarian tumors Fig. 3.

**Table 2:** Univariable and multivariable analyses of factors on frequency of FPS positivity

Parameter	Univariable analysis		Multivariable analysis	
		*p* value	Adjusted odds ratio (95% CI) **	*p* value
Grade of ovarian tumors	% (n)			
Benign (n = 146)	66.4 (97)	< 0.001	1.581 (0.681–3.670)	0.078
Borderline (n = 51)	54.9 (28)			
Malignant (n = 79)	22.8 (18)			
Age	median (range)			
FPS negative	44.0 years (15–54)	< 0.001	0.912 (0.880–0.944)	< 0.001
FPS positive	34.0 years (14–54)			
Tumor size	median (range)			
FPS negative	110.0 mm (16–375)	< 0.001	0.979 (0.972–0.986)	< 0.001
FPS positive	57.0 mm (7–191)			
MRIST	median (range)			
FPS negative	3.00 mm (1.25–7.00)	< 0.001	0.749 (0.609–0.920)	0.006
FPS positive	2.20 mm (2.00–7.00)			
MRI magnetic field strength	% (n)			
1.5 T (n = 198)	46.5 (92)	0.005	1.133 (0.554–2.320)	0.732
3.0 T (n = 78)	65.4 (51)			

FPS, follicle preserving sign; MRIST, Magnetic resonance imaging slice thickness

The results of the univariate and multivariate analyses are displayed in Table 2. In the univariate analysis, FPS was also significantly more common in younger patients, as well as in those with smaller tumor size, thinner MRIST (*p* < 0.001 for all), and high MRI magnetic field strength (p = 0.005). The mean tumor sizes in benign, borderline, and malignant groups were 61.0 mm (range, 7–375 mm), 85.0 mm (range, 16.0–300 mm), and 117.0 mm (range, 22.0–370.0 mm), respectively. The mean tumor size in the benign group was significantly smaller than that in the other groups (*p* < 0.001). The mean patient ages were 36 years (range, 14–54 years), 38 years (range, 17–54 years), and 46 years (range, 18–53 years), respectively. The mean age of the patients in the malignant group was significantly higher than that in the other groups (*p* < 0.001). The mean MRIST was 2.50 mm (range, 1.25–6.50 mm) in the benign group, 3.00 mm (range, 2.00–7.00 mm) in the borderline group, and 3.00 mm (range, 1.25–7.00 mm) in the malignant group. The mean MRIST in the benign group was significantly lower than that in the other groups (*p* < 0.001).

In multivariate analysis, patient age, lesion size, and MRIST were significant factors influencing the frequency of FPS (*p* < 0.05 for all). The odds ratio of the MRIST for the frequency of FPS was 0.749 (95% CI: 0.609–0.920), which was the highest of the three items. Hence, MRIST was the most influential factor impacting the incidence of FPS. To clarify which factors influence MRIST, we performed a multivariate analysis including all variables except MRIST (Table 3). Lesion grade and MRI magnetic field strength were significant factors influencing the MRIST scores (*p* = 0.001 and *p* < 0.001, respectively). The partial regression coefficient for MRI magnetic field strength on MRIST was − 0.831 (95% CI: − 1.903— − 0.568), representing the strongest association.

**Table 3:** The effect of variables associated with MRIST on the frequency of FPS positivity

	Partial regression coefficient	Standard partial regression coefficient	*p* value	95%CI of partial regression coefficient
Grades	0.380	0.205	0.001	0.154 – 0.606
Tumor diameter	0.001	0.059	0.318	− 0.001 – 0.004
Age	− 0.004	− 0.028	0.623	− 0.001 – 0.004
MRI magnetic field strength	− 0.831	− 0.348	< 0.001	− 1.093 – − 0.568

FPS, follicle preserving sign; MRIST, Magnetic resonance imaging slice thickness

When stratified using MRIST, the FPS was significantly more frequent in the benign group than in the malignant group (Table 4). For each lesion grade, a thinner MRIST corresponded to a higher observation rate of FPS sign; however, the incidence of FPS in the benign and borderline groups remained relatively high, even when MRIST exceeded 6 mm (Table 4).

**Table 4:** Relationship between grade level and frequency of FPS positivity under MRIST stratification

MRIST	No. of benign cases (%)	No. of borderline cases (%)	No. of malignant cases (%)
6 mm ≦	6/12^a^ (50.0%)	4/12 (33.3%)	1/20^a^ (5.0%)
3 mm≦ < 6 mm	23 /44^b^ (52.3%)	11/22 (50.0%)	7/34^b^ (20.6%)
< 3 mm	68/90^c^ (75.6%)	13/17 (76.5%)	10/25^c^ (40.0%)

FPS, follicle preserving sign; MRIST, Magnetic resonance imaging slice thickness

There were statistically significant differences between a (*p* = 0.013), b (*p* = 0.012), c (*p* = 0.002)

The histological diagnosis of each ovarian tumor is summarized in Table 5. In addition, the relationship between histological subtype and FPS frequency was evaluated, and the results are summarized in Table 5. When defining a low incidence of FPS as < 30% and a high frequency as > 70%, mucinous cystadenomas demonstrated a low incidence within the benign group. In the borderline malignancy group, serous/seromucinous and endometrioid borderline tumors were associated with a high incidence of FPS. Conversely, adult-type granulosa cell tumors/sertoli cell tumors exhibit a low incidence of FPS. Immature teratomas had the highest incidence of FPS in the malignant group. Mucinous tumors were distinguished by significantly larger tumors (112 mm, range, 53–375) compared to other benign tumors. Serous/seromucinous borderline tumors were smaller in size (58 mm; range, 16–165) and were diagnosed in younger patients (35 years; range, 21–52) compared to other borderline tumors. Mucinous borderline tumors were larger (147 mm, range, 80–300) compared to the size of other borderline malignant tumors. Among malignant tumors, immature teratomas were associated with a significantly younger age (21.5 years, range, 18–25). The FPS positivity rates for other tumors were as follows: 60% for serous/seromucinous cystadenomas, 62.5% for fibromas/thecomas, 40% for struma ovarii, 0% for carcinoid/squamous cell carcinoma, 7.7% for serous carcinoma, 50% for dysgerminoma, 36.4% for metastatic tumors, and 31.3% for mucinous borderline tumor.

**Table 5:** Relationship between histological type and incidence of FPSP

High Incidence (> 70%) of FPSP	n	%	Intermediate incidence (30% ≦70%) of FPSP	n	%	Low Incidence (< 30%) of FPSP	n	%
Benign			Benign			Benign		
Endometriotic cyst	41/51	80.4	Serous/Seromucinous cytadenoma	3/5	60.0	Mucinous cystadenoma	2/17	11.8
Mature teratoma	44/60	73.3	Fibroma/Thecoma	5/8	62.5			
			Struma ovarii	2/5	40.0	Borderline		
Borderline						Adult-type granulosa cell tumor/Sertori cell tumor	0/6	0
Serous/Seromucinous borderline tumor	21/26	80.8	Borderline			Carcinoid tumor	0/1	0
Endometrioid	2/2	100	Mucinous borderline tumor	5/16	31.3			
						Malignant		
Malignant			Malignant			Clear cell carcinoma	4/17	23.5
Immature teratoma	2/2	100	Dysgerminoma	1/2	50.0	Endometroid carcinoma	4/14	28.6
			Metastatic tumor	4/11	36.4	Serous carcinoma	1/13	7.7
			Mixed cell carcinoma	2/5	40.0	Mucinous carcinoma	0/12	0
						Squamous carcinoma	0/3	0

FPSP, follicle preserving sign positivity

## Discussion

Our study found that the malignancy of ovarian tumors is associated with the incidence of FPS. FPS has traditionally been considered to correlate with tumor malignancy, and our findings supported this notion [10]. However, MRIST strongly influences FPS incidence, our results also highlight the importance of considering MRI acquisition parameters when using the presence or absence of FPS to assess tumor malignancy.

Our findings revealed that FPS was significantly associated with patient age, tumor size, MRI magnetic field strength and MRIST, with MRIST being the most influential factor. At the same time, our results indicated that MRIST plays an important role in the detection of FPS. Importantly, we also discovered that MRI magnetic field strength and tumor malignancy significantly affected MRIST. As expected, increased magnetic field strength leads to a thinner MRIST. In contrast, this is likely attributable to the fact that malignant cases tended to require thicker MRIST, possibly due to factors such as tumor size, peritoneal dissemination, and the presence of ascites. Therefore, the previously observed association between FPS and tumor malignancy may have been confounded by the thicker MRIST used in malignant cases.

Even after standardizing MRIST across cases, the frequency of FPS positivity continued to vary according to tumor malignancy. Notably, across all MRIST values, a statistically significant difference in FPS positivity was observed between benign and malignant tumors. One possible explanation is that malignant tumors more aggressively invade the ovarian stroma than benign tumors, leading to follicular loss. Based on our findings, when the MRIST is 6 mm or greater and FPS is negative, the ovarian tumor is highly likely to be malignant. In contrast, when the MRIST is less than 3 mm and FPS is positive, the tumor is more likely to be benign, although the possibility of malignancy cannot be ruled out. In such cases, the malignancy of the ovarian tumor should be assessed in conjunction with other imaging findings. Therefore, the interpretation of FPS in ovarian tumors should always take MRIST into account.

In addition, FPS positivity decreased with age, which may reflect an inverse relationship between age and ovarian function. As the ovarian function declines with age [11], the likelihood of detecting follicles on MRI also decreases accordingly. Tumor size was another significant factor; large tumors were associated with a reduced frequency of FPS. This is likely caused by mechanical stretching of the ovarian stroma by large tumors, which displaces follicles toward the periphery of the ovary, making them less visible on MRI.

Interestingly, this study demonstrated that the frequency of FPS positivity varies according to the histological type of the ovarian tumor. In many cases, this variability appears to be influenced by the inherent characteristics of the tumors themselves. For instance, in serous and seromucinous borderline tumors, the relatively young age of patients and the small tumor size may have contributed to the high rate of FPS positivity. Similarly, among malignant tumors, all immature teratomas exhibited FPS positivity, which may also be attributed to the age of the affected patients. In contrast, mucinous tumors tended to be FPS-negative regardless of their malignant status. This may be explained by their typically large size, which results in a thick MRIST and subsequently reduces the likelihood of detecting the FPS. Additionally, the large size of mucinous tumors may compress the ovarian stroma and consequently render follicles less visible on MRI.

However, certain histological types cannot be adequately explained by patient age, tumor size, MRIST, or tumor malignancy. Notably, FPS was not identified in any of the borderline sex cord-stromal tumors, including adult-type granulosa cell and sertoli cell tumors. In cattle, granulosa cell tumors have been reported to induce ovarian atrophy via the suppression of follicle-stimulating hormone production [12, 13]. The underlying reason is unclear, since it is not established whether a similar mechanism exists in humans. In this study, the investigation of histological type-specific trends remains incomplete. Further studies with large sample sizes are required to validate and expand upon these findings.

This study has certain limitations. First, a bias existed in the distribution of the pathological subtypes. Not all histological types of ovarian tumors were represented in this study, as the prevalence is inherently skewed toward certain histological types, leaving rare forms underrepresented. Therefore, further investigations are needed to evaluate a comprehensive range of tumor types. Second, the presence of normal follicles on the side affected by the tumor was not confirmed through pathological examination. As this study was conducted retrospectively, the presence or absence of follicles was probably not specifically assessed during routine pathological examinations. Third, the scan parameters of the MRI scanners varied, as this was a retrospective, long-term study. Finally, the sample sizes differed among groups, and no validation study was performed. To address these limitations, a large-scale prospective study is required to confirm and expand upon our findings.

In conclusion, tumor malignancy was associated with FPS. The frequency of FPS positivity was influenced by patient age and tumor size; however, the most influential factor was MRIST. While thick slices (≥ 6 mm) reduce the detectability of FPS, malignant tumors were still more likely to lack FPS than benign tumors. Conversely, under thin-slice conditions (< 3 mm), the presence of FPS strongly supports a benign diagnosis, although malignancy cannot be entirely excluded. Furthermore, the frequency of FPS positivity varied according to histological subtype, being relatively high in serous/seromucinous borderline tumors and immature teratomas, but consistently low in mucinous tumors irrespective of malignancy status. Therefore, FPS should always be interpreted in conjunction with MRIST, tumor histology, and other imaging findings when assessing ovarian tumor malignancy.
